# Cutaneous Ovarian Carcinoma Metastases: Case Report and Literature Review

**DOI:** 10.7759/cureus.44459

**Published:** 2023-08-31

**Authors:** Han Li, Milbrey Parke, Marjorie Montanez-Wiscovich

**Affiliations:** 1 Dermatology, University of Florida College of Medicine, Gainesville, USA

**Keywords:** skin manifestations, ovarian epithelial carcinoma, ovarian neoplasms, skin carcinoma, metastases

## Abstract

Metastatic cutaneous ovarian carcinoma is a rare diagnosis with a poor prognosis. Cutaneous manifestations are variable in size and morphology. We report a woman presenting with cutaneous ovarian metastases mimicking reticulated dermatoses. Our patient presented with a four-month history of a mildly pruritic eruption in the setting of stage IV ovarian adenocarcinoma, for which she was undergoing carboplatin, doxorubicin, and bevacizumab chemotherapy. On exam, she had erythematous, indurated papules and plaques involving the right flank and breast, as well as a reticulated erythematous patch on the lower abdomen. Cutaneous ovarian metastases have varied presentations. Our case highlights an uncommon manifestation of ovarian metastases and reviews the prior literature.

## Introduction

Cutaneous metastases remain an uncommon diagnosis. For epithelial ovarian carcinoma metastases, cutaneous involvement is seen in only 3.5% of cases [1]. There is a significant amount of variability in clinical morphology, with some ovarian metastases presenting as indurated plaques, vesicular eruptions, or umbilical nodules (the Sister Mary Joseph nodule) [1]. Cutaneous involvement is the presenting symptom in 40% of all metastatic ovarian malignancies [2]. Therefore, prompt recognition is important. Herein, we report a woman with a reticulated, indurated eruption proven to be cutaneous ovarian metastasis and review dermatological manifestations of ovarian metastases [1-17].

## Case presentation

**Figure 1:**
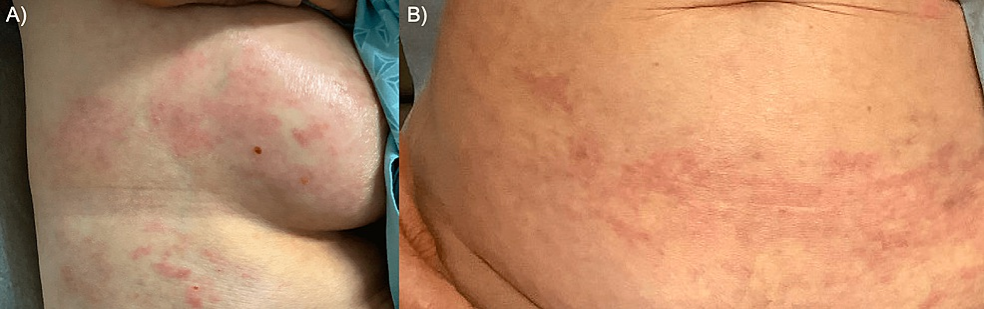
Clinical findings (A) Erythematous indurated papules and plaques on the right breast and flank and (B) reticulated erythematous patches involving the lower abdomen.

A woman in her 60s with a history of stage IV ovarian carcinoma presented with a four-month history of slightly pruritic, indurated papules and reticulated patches on her chest and abdomen. Two years prior to presentation, she presented to an outside hospital with ascites, peritoneal carcinomatosis, and sclerotic bony lesions. Following an ascitic fluid cell block on paracentesis, she was diagnosed with adenocarcinoma of likely ovarian origin, which was treated with five cycles of carboplatin and paclitaxel. A bone biopsy was performed but failed to identify malignancy, and the patient declined a repeat biopsy. A peritoneal biopsy was unable to be performed as the peritoneal carcinomatoses were deemed too small. One year prior to presentation, she transferred to our institution and underwent a laparoscopic biopsy to guide further treatment, which provided the definitive diagnosis of high-grade serous ovarian carcinoma. She was treated with carboplatin, doxorubicin, and bevacizumab chemotherapy. Her rash appeared during her second chemotherapy regimen. Eruption was initially noted on the right breast, extending unilaterally to the right flank and lateral right back. Her primary care physician treated her empirically for herpes zoster due to the dermatomal distribution, but the rash did not improve. Over the following few months, the rash became indurated and progressed to involve the bilateral lower abdomen. On exam, the right breast and chest hosted erythematous, indurated plaques (Figure 1A), and the left and right lower abdomens had reticulated, erythematous patches (Figure 1B). A punch biopsy was performed on the right flank, which showed an infiltrating dermal tumor of irregular islands and trabeculae of atypical cells with foci of glandular formation. Cells were positive for cytokeratin 7 and paired box gene 8 and negative for estrogen receptor, confirming the diagnosis of metastatic cutaneous ovarian carcinoma.

Treatment approaches described in the literature [4] include surgical excision with adjuvant chemotherapy, surgical excision alone, chemotherapy alone, or hospice care. Due to the extensive nature of our patient's metastatic disease, she is not a good candidate for surgical interventions, and she was referred to the oncology team for discussion of chemotherapeutic options. At the time of writing, medical management was still being discussed.

## Discussion

Cutaneous metastases of any kind are an uncommon finding but are particularly rare for ovarian carcinoma, occurring in only 3.5% of cases [1]. Paradoxically, cutaneous metastases are often the presenting symptom of ovarian malignancy in dermatology [2]. The prognosis is poor, with a median survival time of four months. Comparatively, cutaneous metastases occur in 5.3% of all malignancies and occur from a primary breast malignancy in 24% of cases [3]. The mechanism of metastasis varies depending on the presentation, with Sister Mary Joseph nodules most frequently spreading contiguously and non-Sister Mary Joseph nodules spreading by direct implantation.

Cutaneous spread has several risk factors. First, histological types such as high-grade serous carcinoma (as was this case), endometroid carcinoma, and clear cell carcinoma have the highest proportion of cutaneous metastases [4]. Second, prior laparoscopic surgery, especially in settings where there are malignant ascites or intraperitoneal metastases, as in our patient, is thought to provide a route of direct implantation [4]. Third, a history of bevacizumab and other anti-vascular endothelial growth factor treatments can also predispose to cutaneous metastases via acquired resistance and subsequent angiogenesis [4].

Unfortunately, the prognosis for patients with ovarian cutaneous metastases is poor. The greatest prognostic factor in survival is the interval time between diagnosis of ovarian carcinoma and manifestation of cutaneous symptoms, with intervals longer than 40 months having a better prognosis [1,5]. Sites of metastases in surgical scars portend a favorable diagnosis, and concomitant metastatic lesions to other sites are associated with poorer mortality [4]. The optimal treatment remains unknown, but chemotherapy, surgical resection, and radiation have been attempted [4].

Cutaneous ovarian metastases have been reviewed (Table 1). Manifestations similar to the present case are rare. The most common presentations are single or multiple nodules [1,6-17]. Other manifestations include zosteriform lesions, keratotic umbilicated papules, and erythematous and hyperpigmented indurated plaques. Indurated or reticulated erythematous plaques similar to the present case have been reported previously on the thigh [7] and chest [15]. Frequent sites of metastases are the abdomen, chest, or prior sites of intraabdominal surgery. Cutaneous involvement was the presenting symptom in only two reports, both of which manifested as a single nodule in the context of a high-grade serous adenocarcinoma [1,12]. Our case highlights an uncommon presentation of cutaneous ovarian metastases and reinforces the importance of a thorough history and prompt biopsy of suspicious rashes in patients with a history of malignancy.

**Table 1:** Comprehensive review of clinical findings of cutaneous metastatic ovarian carcinoma

Author	Age (years)	Histological subtype/presenting symptom?	Cutaneous findings	Site
Cormio et al. [1]	68	Serous G3/No	Multiple nodules	Laparoscopy scar
	33	Endometroid G1/No	Multiple nodules	Chest, arms
	67	Serous G3/Yes	Single nodule	Umbilicus
	68	Serous G3/No	Single nodule	Port scar
	43	Serous G2/No	Multiple nodules	Chest, abdomen
	36	Serous G3/No	Multiple nodules	Chest
	56	Mucinous G3/No	Multiple ulcerated lesions	Groin
	67	Serous G1/No	Herpetiform lesions	Lower abdomen
	37	Serous G3/No	Single nodule	Drainage scar
Yilmaz et al. [6]	69	Serous/No	Erythematous plaques and nodules	Lower abdomen
Lee et al. [7]	49	Clear cell/No	Reticulated erythematous plaques	Thigh and buttock
Abbas et al. [8]	42	Serous G3/No	Keratotic umbilicated papules	Groin
Cheng et al. [9]	67	Serous/No	Ulcerated erythematous plaques	Abdomen and thighs
Kothiwala et al. [10]	50	Serous/No	Erythematous macules and multiple nodules	Abdomen
Lalich et al. [11]	31	Endometroid G2/No	Single nodule	Upper arm
Kaur et al. [12]	32	High-grade serous/Yes	Single nodule	Abdomen
Ching et al. [13]	30	Low-grade serous/No	Single nodule	Back
Li et al. [14]	48	Hybrid/No	Multiple nodules	Left thigh
McDonald et al. [15]	24	Mucinous/No	Indurated patches	Chest
Schonmann et al. [16]	48	Serous/No	Zosteriform	Lower abdomen
Ruiz et al. [17]	72	Low-grade serous/No	Multiple nodules	Neck and shoulder

## Conclusions

Cutaneous metastatic ovarian carcinoma is rare but can be a presenting symptom of malignancy. Metastases may present as nodules, papules, or rashes with secondary findings. Suspicious rashes in the setting of malignancy should be biopsied for prompt diagnosis. Early detection of cutaneous metastases can assist in guiding management and patient discussion. This case highlights both the breadth of clinical manifestations of cutaneous ovarian metastases and the importance of considering metastatic disease when evaluating rashes in patients with a history of malignancy.
